# Position Statement of the Brazilian Society of Nephrology on Home Hemodialysis

**DOI:** 10.1590/2175-8239-JBN-2025-0286en

**Published:** 2026-02-13

**Authors:** Fernanda Salomão Gorayeb-Polacchini, Dirceu Reis da Silva, Allison Andrade, Isadora Cartaxo de Sousa Calvo, João Cezar Mendes Moreira, José Carolino Divino-Filho, Patrícia Ferreira Abreu, Paulo Henrique Fraxino, Renato Jorge Palmeira de Medeiros, José A. Moura-Neto, Rosilene Motta Elias

**Affiliations:** 1 Sociedade Brasileira de Nefrologia, São Paulo, SP, Brazil.; 2 Hospital de Base, São José do Rio Preto, SP, Brazil.; 3 Hospital de Clínicas de Porto Alegre, Porto Alegre, RS, Brazil.; 4 Faculdade de Medicina Unisinos, São Leopoldo, RS, Brazil.; 5 Karolinska Institute, Department of Clinical Science, Intervention and Technology, Division of Renal Medicine, Stockholm, Sweden.; 6 Universidade Federal de São Paulo, São Paulo, SP, Brazil.; 7 Faculdade Dinâmica do Vale do Piranga, Ponte Nova, MG, Brazil.; 8 Escola Bahiana de Medicina e Saúde Pública, Salvador, BA, Brazil.; 9 Universidade de São Paulo, São Paulo, SP, Brazil.; 10 Universidade Nove de Julho, São Paulo, SP, Brazil.

**Keywords:** Renal Dialysis, Home Hemodialysis, Renal Insufficiency, Chronic kidney Replacement Therapy, Health-Related Quality of Life, Consensus

## Abstract

Home hemodialysis (HHD) has been increasingly consolidated worldwide as an effective modality of kidney replacement therapy (KRT), associated with greater autonomy, flexibility, and quality of life for patients, in addition to allowing individualized prescriptions with more frequent and/or longer sessions. Despite evidence demonstrating survival rates equal to or superior to those of in-center hemodialysis, HHD remains underutilized globally, including in Brazil. Limiting factors include structural, logistical, financial, and cultural barriers, as well as the absence of specific national regulations. This document presents the position of the Brazilian Society of Nephrology (SBN) on HHD, establishing recommendations for patient eligibility, home and dialysis center selection criteria, responsibilities, and technical safety requirements. Two HHD models are described: the self-care modality, in which patients perform the treatment on their own after intensive and strict training, and the assisted modality, carried out with the continuous presence of a healthcare professional. The position emphasizes the need for an associated dialysis center, structured training, safety protocols, continuous monitoring, and emergency backup. Key aspects such as water quality, supply chain logistics, waste disposal, and environmental sustainability are considered essential. The SBN advocates that adherence to HHD should result from shared decision-making between patients, families, and the multidisciplinary team, formalized through an informed consent document. This position aims to support policies, adequate funding, and regulatory adjustments to enable the practice of HHD in Brazil, ensuring care quality, equity of access, and the safety of patients and professionals involved.

## Introduction

Chronic Kidney Disease (CKD) is increasingly prevalent in Brazil, with estimates indicating that more than 170,000 individuals are on dialysis, according to the Dialysis Survey conducted by the Brazilian Society of Nephrology^1^. Most of these individuals are undergoing hemodialysis (HD) treatment in satellite clinics or hospitals, usually three times per week, with each session lasting three to four hours^1^.

Different therapeutic modalities have been adopted worldwide with the aim of improving the quality of life and survival of dialysis patients. Of particular note are HD regimens such as short daily HD (90 to 120 minutes, five to six times per week)^2^; long HD (six to ten hours, three times per week)^3^, performed mainly during the nighttime^4^; and home hemodialysis (HHD)^5,6^, the latter providing the opportunity for more frequent and longer HD sessions. HHD has been increasingly adopted as a dialysis modality worldwide, with specific operating criteria and intended for patients who meet appropriate eligibility requirements^7,8^. However, current legislation regulating HD practice in Brazil (Ministerial Order GM/MS No. 1675/2018) does not establish specific eligibility criteria or operating and safety standards for HHD.

While sustaining life, dialysis is burdensome and invasive for patients and, due to its complexity, constitutes a high-cost procedure for the healthcare system. HD in dialysis centers has disadvantages, including the burden of patient commuting, inflexible treatment schedules, and HD sessions with high ultrafiltration rates, which increase the risk of hypotension, muscle cramps, and fatigue.

Home dialysis modalities (HHD and peritoneal dialysis) are associated with greater flexibility, autonomy, quality of life, and patient satisfaction with treatment, alongside additional benefits resulting from the potential use of longer or more frequent procedures, compared with modalities performed in dialysis centers^9,10,11,12^.

Although home dialysis modalities are still less commonly used worldwide, several efforts have been implemented around the world to increase their adoption^9,13,14^. The 2021 Kidney Disease Improving Global Outcomes (KDIGO) Controversies Conference on home dialysis recommended aligning policies and resources, as well as encouraging the training and engagement of technical teams involved, in order to facilitate broader access to home-based dialysis^9^.

In Brazil, peritoneal dialysis (PD) is well established, although its use is noticeably lower than that of HD. This may be explained by the lower financial support for this modality, the fact that less than 50% of dialysis centers provide this therapy, and existing cultural barriers^1^. PD has the potential for better cost-effectiveness and reduced care complexity, and should be considered a priority as a home-based modality when clinically eligible. In this document, we will focus solely on HHD.

Recently, the first case of self-care HHD in Brazil was reported, describing the intensive training processes and the significant improvement in the patient’s clinical parameters^15^.

There are several reasons that may explain the low utilization of HHD, related to: the healthcare system (local resources, public policies, current legislation, costs, reimbursement challenges, access to healthcare services, and logistical issues); dialysis centers (cultural beliefs, preferences or lack of knowledge, and training among healthcare professionals); and patients (inadequate home environment, clinical, cognitive, and social limitations, absence of a caregiver, poor functional health literacy, and possible increase in household water and energy costs)^9,16^.

Clinical outcomes for dialysis modalities are generally similar, but each may offer advantages and disadvantages depending on the patient’s clinical and social profile. Therefore, the choice of modality must necessarily be a shared decision between the patient and family members, valuing the patient’s preferences and clinical condition, with a view to maximizing quality of life^9,17^.

In literature, observational and randomized studies conducted in different regions indicate that HHD offers better or similar survival rates compared with in-center HD^18,19,20,21,22,23,24^. One study reported a one-year survival rate of 100%, a five-year survival rate of 83.5%, and a 10-year survival rate of 34.6% among HHD patients with arteriovenous fistula self-cannulation. Cardiovascular disease was the leading cause of death^25^, similar to what has been observed in other studies^22,26,27,28,29,30,31,32,33,34,35,36,37,38,39,40,41^. It is important to mention that not all dialysis patients are candidates for kidney transplantation. In addition, a previous study demonstrated similar survival between HHD and expanded-criteria deceased-donor kidney transplantation^31^.

In Brazil, there are still no comparative studies available between HHD and other types of Kidney Replacement Therapy (KRT). Overall, studies comparing outcomes involving HHD should consider that patients eligible for this modality may not have the same clinical and socioeconomic profile as those treated in dialysis centers, and these data should be reviewed from that perspective.

Most studies involve self-care HHD, which is performed without the presence of a healthcare professional. However, HHD can also be carried out with the support of such a professional, although there is little data in the literature on this modality.

In this document, the SBN presents a position statement proposing minimum requirements for the safe and effective practice of HHD in Brazil, both for patients and healthcare professionals, with the aim of contributing to the alignment of public policies, resources, and clinical training within dialysis centers.

## Methods

### Stages in the Development of the Brazilian Society of Nephrology’s Position Statement on Home Hemodialysis

#### Drafting of Recommendations

The recommendations presented in this document were developed based on scientific evidence, current Brazilian legislation, and expert opinion. Members of the SBN Board of Directors, the SBN Dialysis Department, the SBN Professional Advocacy Department, as well as an invited nephrologist with extensive experience in managing HHD programs and a representative from the SBN Patient Committee participated in the drafting of this material.

Situations that resulted in possible differences of opinion had two distinct profiles: (i) technical in nature, in which it was possible to ground the recommendations in specialized literature and current national regulations; and (ii) managerial in nature, in which the suggestions were based on the authors’ experience and the Brazilian context.

After the initial draft was prepared, the text underwent a two-stage consultation and revision process.

#### First Stage of Consultation

The preliminary document was submitted for review by the SBN Board of Directors, the SBN Dialysis Department, and the SBN Professional Advocacy Department. Simultaneously, it was forwarded to the 19 active SBN Regional Offices (Alagoas, Bahia, Ceará, the Federal District, Espírito Santo, Goiás, Maranhão, Minas Gerais, Mato Grosso, Pará, Paraíba, Paraná, Pernambuco, Piauí, Rio Grande do Norte, Rio Grande do Sul, Rio de Janeiro, Santa Catarina, and São Paulo), with the aim of ensuring the representativeness of the country’s diverse regional contexts.

#### Second Stage of Consultation – Public Consultation

The contributions received during the first stage were analyzed and incorporated when appropriate, following joint deliberation by the authors. The updated version was then submitted for Public Consultation, widely publicized on the SBN’s website and social media pages. Concurrently, it was forwarded to the Federal Council of Medicine (CFM) (SEI n^0^ 25.0.000006143-6) and the Brazilian Association of Nephrology Nursing (SOBEN).

## Results

During the Public Consultation, 32 contributions were received, of which 14 (43.7%) expressed full agreement, 14 (43.7%) partial agreement, and 4 (12.5%) disagreement. Of the 32 contributions, there were 42 proposed amendments to the document, of which 32 (76.1%) were incorporated into the final version. The Public Consultation contributions, along with the authors’ respective considerations, are available upon request to the SBN with reasonable justification. The names of the contributors to the Public Consultation were removed, following the recommendation of the SBN Legal Department.

## Discussion

### Definition, Eligibility, Technical Aspects of Home Hemodialysis Treatment and Training

#### Definition and Types of Home Hemodialysis

HHD is defined as HD performed at the patient’s home or in the long-term care facilities for the elderly where the patient resides.

**Figure 1:**
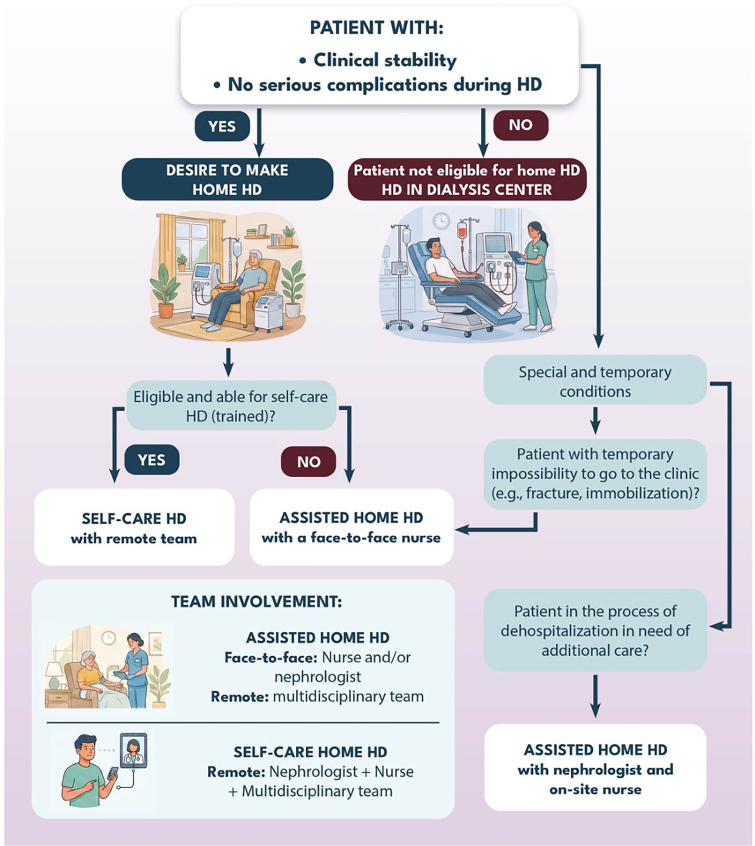
Flowchart of the type of home hemodialysis according to the patient’s clinical scenario.

Types include:

- **Self-care:** when the patient performs the treatment, with or without the assistance of a family member/caregiver, after undergoing intensive and strict training (detailed below in “Intensive training of the patient for self-care home hemodialysis”), and without the presence of a healthcare professional.
- **Assisted:** the patient undergoes treatment in the presence of a healthcare professional, who remains on-site throughout the procedure. The type of professional required depends on the patient’s profile, as described in Figure 1.

Abbreviation – HD: hemodialysis.

#### Eligibility Criteria for Home Hemodialysis

Patients’ enrollment in the HHD program must comply with the requirements listed below and be based on an eligibility assessment conducted by the multidisciplinary team (including at least a nephrologist, a nurse specializing in nephrology, a psychologist, and a social worker), with appropriate documentation in the medical record, considering the beliefs, desires, and expectations of the patient and their family members^38^.

#### Patient Eligibility for Home Hemodialysis

- Patients with CKD and an indication for KRT who choose the HHD modality, with the agreement of family members and the nephrologist, supported by the dialysis center’s multidisciplinary team (at least a nurse specializing in nephrology, a psychologist, and a social worker) and signing the Informed Consent Form (suggested in Appendix 1).
- Being registered with a dialysis center, which will arrange medical appointments at least once a month (at the center or at home) for prescription adjustments.
- Stable clinical condition at the HD center for at least three months, with no complications during sessions that would prevent the safe performance of the procedure.
- Vascular access for HD (restricted to long-term tunneled catheters or arteriovenous fistulas/vascular prostheses) in good condition, i.e., with no evidence of infection or dysfunction.
- Patients who already perform self-cannulation (of arteriovenous fistula or vascular prosthesis) are potentially good candidates for the method, provided that the eligibility criteria and other necessary assessments are met.
- Patients with temporary or permanent mobility limitations and/or those who live far from the dialysis center may be considered for this modality.

If the patient wants to undergo HHD but does not meet the eligibility criteria (related to clinical stability and absence of complications during HD), it is recommended that in-center HD be clearly advised.

In the event that any of the above criteria change over time, indicating excessive risk, the patient should return to in-center dialysis.

#### Eligibility for Each Type of Home Hemodialysis

Together with the multidisciplinary team, the nephrologist must determine which modality is recommended for each patient eligible for HHD: selfcare or assisted.

- **For self-care HHD:** the assessment will be conducted through interviews with the patient, their family members, and any caregivers, in order to analyze learning conditions, acceptability, and interest in this HHD modality.
  - The patient will only be evaluated for the remaining inclusion criteria if the entire multidisciplinary team agrees that they are indeed eligible, at which point training can begin.
  - The patient must have preserved cognitive function and good adherence to treatment.
- **For assisted HHD:** the patient must demonstrate adequate clinical condition, understanding, and motivation for this modality, in addition to meeting clinical, social, and home-related requirements.
- Patients in special and temporary situations may be candidates for assisted HHD, as described in Chart 1 and Figure 1.

**Chart 1:** Suggested type of home hemodialysis and the healthcare professional required, according to the patient’s clinical scenario

Home hemodialysis modality	Patient profile	On-site healthcare professional (minimum)	Remote healthcare professional (minimum)
Self-care	Clinically stable patient, with a history of chronic HD at a dialysis center (satellite or hospital-based) without intercurrences, desire to undergo self-care HHD, eligible, assessed, and with intensive and strict training to perform the procedure.	None	Nephrologist and multidisciplinary team
Assisted	Clinically stable patient, with a history of chronic HD at a dialysis center (satellite or hospital-based) without serious intercurrences, desire to undergo HHD, assessed and eligible. No desire for, or not suitable for self-care home dialysis.	Nurse specializing in nephrology	Nephrologist and multidisciplinary team
	Patient temporarily unable to go to the dialysis center (e.g., fracture, immobilization), with no other changes in clinical status.	Nurse specializing in nephrology	Nephrologist and multidisciplinary team
	Patient being discharged from hospital, not eligible to return to dialysis center.	Nurse specializing in nephrology and a nephrology physician	Multidisciplinary team

Abbreviations – HD, hemodialysis; HHD, home hemodialysis.

The patient profile indications for each type of therapy and the healthcare professional required for assistance are summarized in Chart 1 and Figure 1.

In accordance with current legislation, we emphasize that the SBN does not exempt the presence of a nephrologist for on-site care at dialysis centers, considering the multimorbid profile of patients treated in these settings. With regard to HHD, any change in the clinical scenario requires the patient to be reassessed as to whether the modality should be changed or maintained, thus altering the requirement for the minimum in-person healthcare professional suggested.

#### Eligibility of the Referral Dialysis Center

The dialysis center that develops an HHD program must have the physical infrastructure and human resources required by current national regulations, and must also have:

- An area dedicated to HHD training, separate from other patients undergoing regular HD treatment, allowing for more effective training.
- An individual patient medical record documenting the progress of home-based care provided by the team that performed it, while preserving access to the records at the dialysis center.
- A handbook detailing the HHD program (including Standard Operating Procedures, program indicators, the patient training model, and the Continuing Education system for professionals), as well as a description of the care and administrative team supporting the program.

#### Eligibility of the Home Environment

Before initiating HHD, a technical home visit must be conducted to ensure the feasibility of this activity according to the minimum structural requirements. This visit should be carried out by at least the technical supervisor responsible for water treatment maintenance at the referral dialysis center, a nurse specializing in nephrology, and the social worker. The dialysis center is responsible for assessing the eligibility of the home environment, and it is the institution authorized and supervised for this purpose by the Brazilian Health Regulatory Agency (Resolution - RDC No. 917, dated September 19, 2024, which provides for the Operation of Home Care Services).

- Home access for receiving, transporting, and collecting equipment and supplies should be assessed.
- Cleaning of the water tank and other adjustments may be required to ensure the safe treatment of this input. Its provision should be assessed, including verification of the temperature of the local water supply and the condition of the plumbing system.
- In some cases, adjustments to the electrical system are necessary, as it must be compatible with all equipment to be used and dedicated exclusively to the home setting. Attention should also be given to the availability of accessible means of communication for contacting the dialysis center staff.
- The validation of the residence by the nursing team should include an assessment of the room where the treatment will be performed, with appropriate planning of the layout of equipment and supplies, the area where the patient and healthcare professionals will remain (in the case of assisted HHD), as well as contingency measures for managing intradialytic complications that may require additional interventions. The environment must be safe, thermally comfortable, adequately lit, and free from excessive noise, compatible with regular surface cleaning and with easy access to a sink for frequent hand hygiene. Importantly, household pets must not be allowed in the dialysis area, and the access of young children should be restricted.
- Immediate care must be available through urgent and emergency transport services during the HHD procedure, ensuring that the patient is transferred to the nearest hospital facility, preferably the one designated by the dialysis center.

### Responsibilities

- The technical supervisors (physician and nurse specializing in nephrology) at the referral dialysis center will have the same responsibilities, in accordance with current national regulations, as they do at the dialysis center with regard to HHD.
- Nephrology nurses responsible for training must be dedicated and experienced professionals (with a minimum of six months of experience in HD), competent in all relevant processes, in water treatment, and in adult instruction.
- The technical supervisor for water treatment at the referral dialysis center is responsible for both the initial inspection of the electrical installation and drinking water distribution system, as well as the periodic inspection of the equipment related to water treatment for HHD.
- The nurse specializing in nephrology and the nephrologist must establish, implement, and periodically update Standard Operating Procedures for each HHD flow: patient admission, home assessment, patient and/or caregiver training, supply delivery, machine maintenance control, waste control, registration and storage of medical records and consultation records.
- It is the responsibility of the nephrologist to prescribe HDD, while the nurse specializing in nephrology is responsible for defining the specific parameters of their activities.
- The dialysis center must plan for and ensure patient care in the event of complications related to the HD process, including procedures that guarantee continuity of care whenever necessary.
- The referral dialysis center is responsible for the logistics of supplies delivery and equipment transportation, for providing personal protective equipment, and for arranging the transportation of healthcare professionals, in compliance with the applicable laws and regulations. Additionally, a Health Care Waste Management Plan must be developed, describing the procedures for conditioning, segregation, storage, and final disposal (as required by RDC 222, dated March 28, 2018, and RDC No. 917, dated September 19, 2024). Furthermore, the waste-disposal plan (including sharps, biological, and non-biological waste) must be aligned with the local Health Department.
- The dialysis center should maintain records of the equipment and supplies used in the home setting and be responsible for presenting them to sanitation authorities whenever required, in addition to ensuring traceability and control of preventive maintenance.
- The social work and psychology teams must assess the family structure, patient autonomy, and social and family resources available should they be needed^39^, as well as the impact of implementing HD in the home environment and on family life (both in terms of understanding what usually occurs and potential financial impacts, such as increased water and electricity consumption)^23^.

#### How to Perform Home Hemodialysis

- Any HD or hemodiafiltration (HDF) machine duly registered with ANVISA and equipped with ultrafiltration control may be used; smaller-scale equipment and supplies intended for home use are still awaited in Brazil.
- HHD allows for greater flexibility in prescribing, with the potential use of higher frequency and/or longer treatment sessions. The choice of frequency and duration of hemodialysis sessions should be based on careful clinical assessment and medical prescription, taking into account dialysis adequacy parameters, individual patient conditions, and their preferences, within a shared decision-making process.
- The medical prescription for HHD (especially regarding the choice of blood and dialysate flows, session frequency, and procedure duration) should define the standards to be followed^42,43,44^. It is important to note that: (1) an increase in the total number of weekly HD hours is associated with improved survival; (2) more frequent and longer HD sessions allow for lower ultrafiltration rates, reducing the risk of hemodynamic instability, tissue ischemia, arrhythmias, and sudden death; and (3) avoiding prolonged intervals of no HD is associated with improved survival^6,45,46,47^.
- The frequency of collection and types of laboratory tests must, as a minimum requirement, follow the schedule established by applicable regulations. Attention should be given to hypophosphatemia, which is commonly observed in frequent and long-term HHD^48^.
- The extracorporeal HD system (dialyzers and bloodlines) cannot be reprocessed and must be properly disposed of after use (according to RDC 222, dated March 28, 2018).
- For assisted HHD, a nurse specializing in nephrology is responsible for systematically assessing the patient’s general condition, complaints, blood pressure, heart rate, and temperature. If any abnormalities are identified, the nurse must notify the physician before initiating the session (including systolic blood pressure – SBP < 90 mmHg; SBP > 180 mmHg; tachycardia/bradycardia; hypo/hyperthermia; changes in general condition; or clinical complaints).
- Blood pressure monitoring: for HD sessions lasting up to four hours and performed during the patient’s waking hours, it is recommended that blood pressure be measured every 30 to 60 minutes during the session. For patients undergoing nocturnal self-care HHD, there is no requirement for blood pressure measurement every 30 minutes; it is suggested that measurements be taken at least at the beginning and at the end of the session.
- The patient (in the case of self-care HHD) or the healthcare professional must keep a record, at every dialysis session, of the following data: blood pressure, ultrafiltration volume, pre and post-HD weight, time of start and end of therapy, any intercurrent events, and the corresponding interventions. Blood pressure and heart rate measurements must be documented in the patient’s medical record.
- It is recommended that remote telemonitoring (performed by the team from the patient’s referral dialysis center) occur regularly and whenever necessary or advisable. In the case of self-care HHD, it is suggested that this monitoring be conducted at least during the first two weeks of treatment. After this period, the patient should continue follow-up with a multidisciplinary team and be evaluated according to individualized needs, in addition to the regularly scheduled appointments.
- Remote consultations provided by a nephrologist in urgent/emergency cases and with demonstrable impediment to in-person care are valid in accordance with Article 37, Chapter V of the 2019 Code of Medical Ethics. In addition, the regulation of teleconsultation in Brazil is established by Law No. 14,510/22 and CFM Resolution No. 2,314/2022.
- Pre-defined backup services for urgent/emergency care are required, and HHD should not be performed in locations where such support is unavailable. Emergency care and transport/transfer should be provided by the Mobile Emergency Care Service (SAMU) or by a contracted ambulance service, preferably with an average response time of 30 minutes.
- Outpatient consultations should be scheduled at least once a month and provide patients with access to a nephrologist, a nurse specializing in nephrology, a psychologist, a social worker, and a nutritionist.
- The patient may return to the dialysis facility whenever they choose or whenever deemed necessary by the care team, for reassessment and, if indicated, for further training. The dialysis center should establish mechanisms to enable the patient to resume in-center HD, provided that there is clinical necessity or if the patient elects to discontinue the home modality. This should be done in accordance with the patient’s clinical condition and subject to slot availability.
- Should any questions arise, the patient should contact the nephrology nurse or nephrologist at the dialysis center by phone, during regular operating hours. Patient training should include the provision of a card containing the telephone numbers of the dialysis center.
- In the event of problems with the dialysis machine that cannot be resolved in time for the next session, the patient may undergo HD treatment at the center, at a timeslot to be agreed upon with the scheduling department.
- Regular home visits should be scheduled monthly, or more frequently if necessary, by the technicians responsible for water treatment and HD machine maintenance.
- Measures to ensure patient safety in the HHD modality^8^, must be clearly defined, including the incorporation and maintenance of a Safety Culture^49^, the establishment of barriers to prevent adverse events^50^, and the investigation and management of incidents by the Patient Safety Committee of the dialysis center responsible for the treatment^51^.
- Recommendations regarding immunizations must comply with the National Immunization Policy^52^ of the Brazilian Ministry of Health.
- The care of patients with hepatitis B follows the same protocol as established by current national regulations, and it is recommended that the procedure be performed using equipment dedicated exclusively to the patient and by healthcare workers with protective antibody levels (anti-HBs).
- Sustainable management^53,54^ of reject water is recommended, compatible with domestic use for garden irrigation, general cleaning, laundry, and toilet flushing, as well as careful disposal of dialysate effluent in accordance with applicable legislation.
- The disposal of HHD supplies must comply with the same standards applicable to satellite dialysis centers, with bloodlines and dialyzers discarded as biological waste. Sharps must be placed in appropriate containers and disposed of through proper channels. It is also recommended that, during HHD training, patients be informed about where waste is disposed of and how it is transported. Under no circumstances should waste be discarded as regular household garbage.

#### Intensive Training of Patients for Self-Care Home Hemodialysis

The physical environment required to begin patient training may be the same environment in which they undergo routine HD, provided that greater privacy is ensured.

The patient will be informed about how the HD treatment process will take place at their home and that, in the event of any difficulties (whether related to supplies or the company involved), they are entitled to return to in-center HD at the referral center.

The patient will be trained in procedures related to HD itself: preparing, turning on and shutting down the machine, understanding and resolving the various alarms on the equipment, applying safety procedures, and performing arteriovenous fistula cannulation or catheter connection.

The patient will be monitored and trained from the outset by the same nurse, at a pre-determined time, according to their therapy routine. The training comprises a minimum period of two months or 24 sessions, which may be extended as necessary. A home contact person will be trained to recognize alarms and adverse events^50^ and perform the relevant procedures; this caregiver may also receive comprehensive training together with the patient.

Patient (and caregiver) training for HHD will consist of the following stages:

1. The initial component consists of an introduction and guidance for the patient, addressing independence and responsibility with treatment, as well as vascular access care and the importance of strictly following the safety routine at home.
2. The teaching techniques used during training are the responsibility of the dialysis center and may include practical examples demonstrated repeatedly. When applied throughout the training period, this process builds confidence in operating the HD or HDF machine, troubleshooting alarms, and assembling and disassembling the equipment required for dialysis. It also enables the patient to learn safe self-cannulation of the vascular access or connection of the central venous catheter, while empowering them to discontinue treatment, use the machine’s disinfection technology, and perform water treatment procedures.
3. The training includes troubleshooting potential nighttime problems and emergency protocols, including initiating an emergency call to 192 (or to a private ambulance service, in the case of patients covered by supplementary health providers rather than the SUS) or heading to the nearest emergency department.
4. A handbook containing all the content covered during the training should be provided to the patient, reviewing the precautions for initiating, maintaining, and terminating treatment, as well as how to respond to alarms and possible emergency procedures.
5. During the training period, three written tests should be administered to ensure that the patient and/or caregiver are able to perform the procedures safely. These written exams are a way to formalize the effectiveness of the training, confirming that the patient and/or caregiver know how to proceed when faced with various situations—whether risky or not—that may occur at home. A minimum score of 70% to 90% (as determined by the dialysis center) on these tests is suggested. Training should cover defined topics, problem-solving skills, and competency-based assessments.
6. At the end of the training period, the patient and/or caregiver is expected to be able to perform the entire HD process without any assistance from the nursing staff (who remain only as observers in the dialysis room where the training takes place). This safety step ensures that all staff can observe the patient and/or caregiver as if they were in an HHD session. The duration of this period may vary from patient to patient.
7. After the training period, the patient and/or caregiver should be able to perform all procedures in practice, at which point they will be cleared by the nephrologist and the nurse specializing in nephrology to begin treatment at home. If there is any concern about the execution of the procedure—whether from the perspective of the nurse, the physician, or the patient and/or caregiver—the patient’s dialysis treatment should be adjusted to another form of dialysis that is more appropriate for each context.
8. The patient should be instructed to bring to at least monthly appointments a record sheet (provided by the team) containing HHD information, such as weight, blood pressure, and the ultrafiltration rate schedule performed by themselves. It is the patient’s responsibility to present this record document at their monthly visits for subsequent filing in their medical records.

## Technical Aspects of Water Quality, Cost Analysis, and Legal Aspects of Home Hemodialysis

### Water Treatment for Home Hemodialysis

Water treatment must comply with the parameters established in current national regulations. The dialysis center designated as the reference facility for the HDD program must keep water analysis reports available for submission to the Health Regulatory Agency.

- In accordance with ANVISA RDC No. 11, dated March 13, 2014, the domestic water supply to be treated with portable reverse osmosis (PRO) equipment must meet drinking water standards as established in current legislation (Art. 45). Its physical and organoleptic characteristics (apparent color, turbidity, taste, odor, free residual chlorine, and pH) must be measured before each HHD session (according to Chart I of the annex to the aforementioned RDC), with a record in the medical chart.
- The water produced by the PRO must meet the same standards established for water quality (according to Chart II of the annex to ANVISA RDC No. 11, dated March 13, 2014, which sets forth the components for monthly and semi-annual monitoring). The analyses must be performed by an analytical laboratory licensed by the competent sanitation agency.

In detail, the following are recommended:

- Cleaning the water tank at initiation and every six months thereafter;
- Osmosis: sanitization every 30 days for HD and every 15 days for HDF;
- Daily testing of water chlorine levels before the start of the HD session;
- Monthly water analysis for microbiological testing;
- Semi-annual water analysis for physical-chemical testing;
- Annual dialysate analysis;
- Annual drinking water analysis;
- Maintenance of equipment for possible replacement of valves, pumps, couplings, and hoses, if necessary, and whenever a malfunction occurs.

In the case of multifamily buildings, such as apartments and condominiums, contracting and supervising the cleaning of reservoirs (water tanks) by a specialized company is the responsibility of the building manager. In addition to the initial and semi-annual routine, extra cleaning is mandatory in situations of potential contamination (water main ruptures, debris entering the system, among others). A prior alignment meeting with the building manager is suggested and, if necessary, with the presence of the nephrologist, the nurse specializing in nephrology, and/or the technical supervisor responsible for water treatment at the dialysis service.

### Cost and Funding of Home Hemodialysis

The cost should include the use of essential supplies and dialysis and reverse osmosis machines. The supplies are the same as those used in dialysis centers and include single-use dialyzers and arterial and venous blood line sets, acidic and basic dialysis solutions, pressure isolators, fistula needles, sanitizer for machine disinfection, filters used in the disinfection process, saline solution, gauze, heparin, gloves, and syringes.

Reimbursement by paying sources—both in the public system and in supplementary health care—should prioritize a balance between cost and benefit for providers and dialysis centers, respecting local conditions and needs. The discussion of funding models is essential to make HHD feasible in Brazil. There must be a clear definition of eligibility criteria and requirements to be met in order to preserve quality and safety, with proper cost assessment and adequate definition of reimbursement amounts.

### Legal Aspects of Home Hemodialysis

Currently, Brazil does not have specific regulations for HHD—such as an RDC or ordinance from the Ministry of Health—that define standards for eligibility, execution, evaluation, and monitoring of this therapeutic modality.

In the Resolution that provides for the Operation of Home Care Services (Resolution–RDC No. 917, of September 19, 2024), Article 25 states that the Home Care Service, in accordance with the Home Care Plan, must ensure the provision of dialysis therapy and that, when performing hemodialysis, the dialyzer must be single-use.

Therefore, this position statement from the SBN aims to establish preliminary parameters for HHD in Brazil, preserving the quality and safety of the procedure, as well as the performance of the professionals involved, in addition to aligning HHD with the legislation pertaining to the specialty. Current regulations issued by ANVISA and the Brazilian Ministry of Health should be adjusted to allow for home hemodialysis treatment and stipulate best practices for its implementation.

## Conclusion

Adherence to HHD modality should therefore be the subject of joint reflection among the healthcare team, the patient, and their family members, resulting in a formal therapeutic plan and the signing of an informed consent form, in which the autonomous choice of modality is declared and the expectations and responsibilities of each party involved are outlined.

This document provides technical and operational recommendations aimed at establishing a foundation for the safe, effective, and feasible practice of this modality in Brazil. By integrating clinical, logistical, legal, and sustainability aspects, this position statement from the Brazilian Society of Nephrology aims to support agreements between managers and practitioners, both in the SUS (Brazilian Unified Health System) and in supplementary health care. It further aims to contribute to expanding access and consolidating policies aligned with international quality standards in dialysis.

## Data Availability

The contributions to the Public Consultation and the respective considerations of the authors may be obtained upon formal request to the Brazilian Society of Nephrology (SBN), accompanied by reasonable justification. On the advice of the SBN Legal Department, the names of the contributors have been suppressed.

## Supplementary Material

The following online material is available for this article:

Appendix 1 Template for informed consent form for home hemodialysis or hemodiafiltration.
