# Supplementary material for: Position Statement of the Brazilian Society of Nephrology on Home Hemodialysis
Source: J Bras Nefrol. 2026 Feb 13;48(1):e20250286. doi: 10.1590/2175-8239-JBN-2025-0286en (PMC12904603; doi:10.1590/2175-8239-JBN-2025-0286en)
Supplement: Appendix 1 [file 2175-8239-jbn-48-1-e20250286-suppl1.pdf]

Braz. J. Nephrol.
https://doi.org/10.1590/2175-8239-JBN-2025-0286en

Supplementary Material to "Position Statement of the Brazilian Society of Nephrology on Home Hemodialysis"

INFORMED CONSENT FORM FOR HOME HEMODIALYSIS/HEMODIAFILTRATION

Patient Name: ____

Date of birth:____/____/________

Medical Record Number: _________________________

Address:____

1. Purpose of the processing

I have been informed that I have chronic kidney disease and that I continuously need dialysis treatment. I was advised about the possibility of performing hemodialysis in a home environment, provided that the medical, technical and structural criteria were met.

2. Procedure

Home hemodialysis consists of carrying out dialysis treatment at my home, with the use of appropriate equipment and materials. The treatment requires discipline, responsibility and an appropriate environment, according to technical guidelines provided by the dialysis center's care team.

In case I opt for self-care hemodialysis, without the presence of the health professional in person, I must undergo intensive and rigorous training and be released by the medical and multiprofessional team of my dialysis clinic.

3. Expected benefits

- Greater comfort and autonomy

- Flexibility in session schedules

- Reduced travel to clinics or hospitals

- Improved quality of life

- Easier to adjust the prescription to the patient's needs and preferences

4. Risks and possible complications (likely to occur both in the dialysis center and in the home context):

I was fully informed about the risks inherent to performing hemodialysis at home, and trained on its prevention, detection and management, which include, but are not limited to:

· **Clinical risks:**

- Local infection and/or bloodstream infection associated with vascular access (fistula, catheter)

- Hypotension (low blood pressure) during or after the session

- Hypertension (high blood pressure)

- Cardiac arrhythmias

-Bleeding

- Thrombosis of vascular access

- Air embolism

· **Technical risks:**

- Equipment malfunction or failure

- Accidental disconnections of the venous or arterial line

- Improper programming of the dialysis machine

- Contamination of materials or solutions

- Problems with the supply of electricity or treated water

- Errors in the manipulation of the devices by the patient or caregiver

· **Structural and environmental risks, arising from:**

- Qualitative and quantitative problems in the supply of drinking water

- Lack of adequate environment (hygiene, ventilation, space)

- Inadequate storage of inputs and materials

- Damage to the regularity and stability of the power grid

- Delay or absence of delivery of essential inputs

- Difficulty in quickly accessing urgent care

In addition, since this is a self-care therapy, I am aware that the absence of continuous face-to-face medical supervision may eventually delay the identification of serious complications, which makes constant vigilance and adequate training mandatory.

5. Patient and/or caregiver responsibilities

- Correctly follow the protocol and training provided by the health team

- Keep the environment clean, safe and functional

- Notify any clinical change immediately

- Ensure the presence of a trained caregiver, when indicated

- Preserve and properly operate equipment and supplies

- Maintain the availability of telephone contact with the care team

6. Available Alternatives

I was informed about the existence of other modalities of renal replacement therapy, notably peritoneal dialysis and hemodialysis in a specialized clinic or hospital, which remain available at any time, according to medical decision.

7. Right to refuse or interrupt

I have the full right to refuse or interrupt this treatment, and I may choose another modality without prejudice to my care or the bond with the care team.

8. Declaration of consent

I declare that I have received all the necessary information about the home hemodialysis procedure, including its risks, benefits and alternatives, and that I have had the opportunity to clarify doubts. I therefore authorize the performance of this treatment.

Signatures:

Patient:_____________________________________

Taxpayer Identification Number: _______________________

Date:____/____/_______

Legal guardian (if applicable): __________________________________

Taxpayer Identification Number: _______________________

Date:____/____/_______

Responsible health professional: __________________________________

Regional Council of Medicine: __________________

Date:____/____/_______

Witness 1: ____________________________________

IDENTIFICATION:_______________________

Witness 2: ____________________________________

IDENTIFICATION:_______________________
