# Supplementary material for: Position Statement of the Brazilian Society of Nephrology on Home Hemodialysis
Source: J Bras Nefrol. 2026 Feb 13;48(1):e20250286. doi: 10.1590/2175-8239-JBN-2025-0286en (PMC12904603; doi:10.1590/2175-8239-JBN-2025-0286en)
Supplement: Anexo 1 [file 2175-8239-jbn-48-1-e20250286-suppl2.pdf]

Braz. J. Nephrol.
https://doi.org/10.1590/2175-8239-JBN-2025-0286pt

Material Suplementar para "Posicionamento da Sociedade Brasileira de Nefrologia sobre hemodiálise domiciliar"

TERMO DE CONSENTIMENTO LIVRE E ESCLARECIDO PARA HEMODIÁLISE/ HEMODIAFILTRAÇÃO DOMICILIAR

Nome do(a) paciente: ____

Data de nascimento: ____/____/________

Número do prontuário: __________________________

Endereço: ____

1. Finalidade do tratamento

Fui informado(a) de que tenho doença renal crônica e de que necessito continuamente de tratamento dialítico. Fui orientado(a) quanto à possibilidade de realizar hemodiálise em ambiente domiciliar, desde que atendidos os critérios médicos, técnicos e estruturais.

2. Procedimento

A hemodiálise domiciliar consiste na realização do tratamento dialítico em minha residência, com o uso de equipamentos e materiais apropriados. O tratamento exige disciplina, responsabilidade e ambiente adequado, conforme orientações técnicas fornecidas pela equipe assistencial do centro de diálise.

Para o caso de eu optar por hemodiálise de autocuidado, sem a presença do profissional de saúde presencial, devo passar por um treinamento intensivo e rigoroso e ser liberado pela equipe médica e multiprofissional da minha clínica de diálise.

3. Benefícios esperados

- Maior conforto e autonomia

- Flexibilidade nos horários das sessões

- Redução de deslocamentos até clínicas ou hospitais

- Melhora da qualidade de vida

- Maior facilidade de ajuste da prescrição às necessidades e preferências do paciente

4. Riscos e possíveis complicações (passíveis de ocorrência tanto no centro de diálise quanto no contexto domiciliar):

Fui plenamente informado(a) sobre os riscos inerentes à realização da hemodiálise em domicílio, e capacitado(a) quanto à sua prevenção, detecção e manejo, sendo que eles incluem, mas não se limitam a:

- **Riscos clínicos:**

- Infecção local e/ou infecção de corrente sanguínea associada ao acesso vascular (fístula, cateter)

- Hipotensão (pressão baixa) durante ou após a sessão

- Hipertensão (pressão alta)

- Arritmias cardíacas

- Hemorragias

- Trombose do acesso vascular

- Embolia aérea

- **Riscos técnicos:**

- Mau funcionamento ou falha dos equipamentos

- Desconexões acidentais da linha venosa ou arterial

- Programação inadequada da máquina de diálise

- Contaminação dos materiais ou soluções

- Problemas com o fornecimento de energia elétrica ou água tratada

- Erros na manipulação dos dispositivos por parte do paciente ou cuidador

· **Riscos estruturais e ambientais, decorrentes de:**

- Problemas qualitativos e quantitativos no fornecimento de água potável

- Falta de ambiente adequado (higiene, ventilação, espaço)

- Armazenamento inadequado de insumos e materiais

- Prejuízo na regularidade e estabilidade da rede elétrica

- Atraso ou ausência de entrega de insumos essenciais

- Dificuldade de acesso rápido a atendimento de urgência

Além disso, uma vez que se trata de uma terapia de autocuidado, estou ciente de que a ausência de supervisão médica presencial contínua pode eventualmente atrasar a identificação de complicações graves, o que torna mandatória vigilância constante e capacitação adequada.

5. Responsabilidades do paciente e/ou cuidador

- Seguir corretamente o protocolo e o treinamento fornecido pela equipe de saúde

- Manter o ambiente limpo, seguro e funcional

- Notificar qualquer alteração clínica imediatamente

- Garantir presença de cuidador treinado, quando indicado

- Preservar e operar corretamente os equipamentos e insumos

- Manter a disponibilidade de contato por via telefônica com a equipe assistencial

6. Alternativas disponíveis

Fui informado(a) sobre a existência de outras modalidades de terapia renal substitutiva, notadamente a diálise peritoneal e a hemodiálise em clínica especializada ou hospital, que permanecem disponíveis a qualquer momento, conforme decisão médica.

7. Direito de recusa ou interrupção

Tenho pleno direito de recusar ou interromper este tratamento, podendo optar por outra modalidade sem prejuízo ao meu cuidado ou ao vínculo com a equipe assistencial.

8. Declaração de consentimento

Declaro que recebi todas as informações necessárias sobre o procedimento de hemodiálise domiciliar, incluindo seus riscos, benefícios e alternativas, e que tive a oportunidade de esclarecer dúvidas. Autorizo, assim, a realização do referido tratamento.

Assinaturas:

Paciente: ____________________________________

CPF: ______________________

Data: ____/____/_______

Responsável legal (se aplicável): _________________________________

CPF: ______________________

Data: ____/____/_______

Profissional de saúde responsável: _________________________________

CRM/COREN: __________________

Data: ____/____/_______

Testemunha 1: ___________________________________

RG: ______________________

Testemunha 2: ___________________________________

RG: ______________________
