# Supplementary material for: Position Statement of the Brazilian Society of Nephrology on Home Hemodialysis
Source: J Bras Nefrol. 2026 Feb 13;48(1):e20250286. doi: 10.1590/2175-8239-JBN-2025-0286en (PMC12904603; doi:10.1590/2175-8239-JBN-2025-0286en)
Supplement: Translation

# Translation: Posicionamento da Sociedade Brasileira de Nefrologia sobre hemodiálise domiciliar

**DOI:** 10.1590/2175-8239-JBN-2025-0286pt

**Authors:** Fernanda Salomão Gorayeb-Polacchini, Dirceu Reis da Silva, Allison Andrade, Isadora Cartaxo de Sousa Calvo, João Cezar Mendes Moreira, José Carolino Divino-Filho, Patrícia Ferreira Abreu, Paulo Henrique Fraxino, Renato Jorge Palmeira de Medeiros, José A. Moura-Neto, Rosilene Motta Elias

**Affiliations:** 1 Sociedade Brasileira de Nefrologia, São Paulo, SP, Brasil.; 2 Hospital de Base, São José do Rio Preto, SP, Brasil.; 3 Hospital de Clínicas de Porto Alegre, Porto Alegre, RS, Brasil.; 4 Faculdade de Medicina Unisinos, São Leopoldo, RS, Brasil.; 5 Karolinska Institute, Department of Clinical Science, Intervention and Technology, Division of Renal Medicine, Stockholm, Sweden.; 6 Universidade Federal de São Paulo, São Paulo, SP, Brasil.; 7 Faculdade Dinâmica do Vale do Piranga, Ponte Nova, MG, Brasil.; 8 Escola Bahiana de Medicina e Saúde Pública, Salvador, BA, Brasil.; 9 Universidade de São Paulo, São Paulo, SP, Brasil.; 10 Universidade Nove de Julho, São Paulo, SP, Brasil.

**Keywords:** Hemodiálise, Hemodiálise Domiciliar, Insuficiência Renal Crônica, Terapia Renal Substitutiva, Qualidade de Vida Relacionada à Saúde, Consenso

## Abstract

A hemodiálise domiciliar (HDD) tem se consolidado mundialmente como modalidade eficaz de terapia renal substitutiva (TRS), associada a maior autonomia, flexibilidade e qualidade de vida dos pacientes, além de possibilitar prescrição individualizada com sessões mais frequentes e/ou de maior duração. Apesar de estudos demonstrarem sobrevida igual ou superior à da hemodiálise em clínicas, a HDD permanece subutilizada globalmente, incluindo no Brasil. Entre os fatores limitantes, destacam-se barreiras estruturais, logísticas, financeiras e culturais, além da ausência de regulamentação nacional específica. O presente documento expõe o posicionamento da Sociedade Brasileira de Nefrologia (SBN) sobre a HDD, estabelecendo recomendações para elegibilidade de pacientes, critérios de seleção do domicílio, responsabilidades e requisitos técnicos de segurança. Dois modelos de HDD são descritos: a modalidade de autocuidado, em que o paciente realiza sozinho o tratamento após treinamento intensivo e rigoroso, e a modalidade assistida, realizada sempre com apoio presencial de um profissional de saúde. Este posicionamento reforça a necessidade de vínculo com centro de diálise, treinamento estruturado, protocolos de segurança, monitoramento contínuo e retaguarda de urgência. Aspectos como qualidade da água, logística de insumos, descarte de resíduos e sustentabilidade ambiental são considerados fundamentais. A SBN defende que a adesão à HDD deve resultar de decisão compartilhada entre paciente, familiares e equipe multiprofissional, formalizada por meio de um termo de consentimento livre e esclarecido. O posicionamento busca subsidiar políticas, financiamento adequado e ajustes regulatórios que viabilizem a prática da HDD no Brasil, assegurando qualidade assistencial, equidade de acesso e preservação da segurança de pacientes e profissionais envolvidos.

## Introdução

A Doença Renal Crônica (DRC) tem prevalência crescente no Brasil, estimando-se que mais de 170.000 indivíduos estejam em diálise, segundo o Censo de Diálise da Sociedade Brasileira de Nefrologia^1^. A maioria desses indivíduos está em tratamento de hemodiálise (HD) em clínicas satélites ou em hospitais, em geral três vezes por semana, com duração de três a quatro horas por sessão^1^.

Diferentes modalidades terapêuticas difundiramse mundialmente, com o intuito de melhorar a qualidade de vida e a sobrevida de pacientes em diálise, destacando-se, em especial, esquemas de HD, como a HD curta diária (90 a 120 minutos, cinco a seis vezes por semana)^2^; HD longa (seis a dez horas, três vezes por semana)^3^, realizada principalmente no período noturno^4^; e a hemodiálise domiciliar (HDD)^5,6^, tendo esta última a oportunidade de propiciar condições para sessões de HD mais frequentes e mais longas. A HDD tem sido crescentemente adotada como modalidade de diálise ao redor do mundo, com critérios específicos de funcionamento e destinada a pacientes com elegibilidade adequada^7,8^. Entretanto, a legislação atualmente vigente que regulamenta a prática de HD (Portaria GM/MS nº 1675/2018) não estabelece critérios específicos de elegibilidade e padrões de funcionamento e segurança para a HDD.

Ao mesmo tempo em que mantém a vida, a diálise é onerosa e invasiva para os pacientes, além de constituir, por sua complexidade, um procedimento de alto custo para o sistema de saúde. A HD em centros de diálise apresenta desvantagens, incluindo o ônus do deslocamento para os pacientes, a inflexibilidade dos horários de tratamento e sessões de HD com taxas elevadas de ultrafiltração, o que determina maior risco de hipotensão, câimbras e fadiga.

As modalidades de diálise domiciliar (HDD e diálise peritoneal) estão associadas a maior flexibilidade, autonomia, qualidade de vida e satisfação do paciente com o tratamento, além dos benefícios adicionais decorrentes do potencial emprego de maior duração ou frequência do procedimento, em comparação às modalidades executadas em centros de diálise^9,10,11,12^.

Apesar de a modalidade de diálise domiciliar ainda ser menos utilizada globalmente, vários esforços ao redor do mundo têm sido implementados com o objetivo de aumentar a sua adoção^9,13,14^. A Conferência de Controvérsias do *Kidney Disease Improving Global Outcomes* (KDIGO) sobre diálise domiciliar, realizada em 2021, recomendou o alinhamento de políticas e recursos, além do estímulo à capacitação e ao engajamento das equipes técnicas envolvidas, de modo a facilitar o maior acesso à diálise domiciliar^9^.

No Brasil, a prática de diálise peritoneal (DP) está bem estabelecida, embora haja utilização marcadamente inferior em relação à HD, o que pode ser explicado pelo menor repasse financeiro para essa modalidade, pela oferta da terapia por menos de 50% dos centros de diálise e por barreiras culturais^1^. A DP apresenta potencial de melhor custo-efetividade e menor complexidade assistencial, devendo ser considerada prioritariamente como modalidade domiciliar quando clinicamente elegível. No presente documento, iremos nos deter somente na HDD.

Recentemente, foi descrito o primeiro caso de HDD de autocuidado no Brasil, explicando os processos do treinamento intensivo e a melhora importante de parâmetros clínicos do paciente^15^.

Existem várias razões que podem explicar a baixa utilização da HDD, relacionadas: ao sistema de saúde (recursos locais, políticas públicas, legislação vigente, custos, dificuldade de reembolso, acesso aos serviços de saúde e problemas de logística); às clínicas de diálise (crenças culturais, preferências ou falta de conhecimento e treinamento dos profissionais de saúde); e aos pacientes (ambiente doméstico inadequado, limitações clínicas, cognitivas e sociais, ausência de cuidador, falha no letramento funcional em saúde e eventual aumento do custo de água e energia do domicílio)^9,16^.

Os desfechos clínicos das modalidades de diálise, em geral, são semelhantes, mas cada uma pode oferecer vantagens e desvantagens de acordo com o perfil clínico e social do paciente. Sendo assim, a escolha da modalidade deve necessariamente ser uma decisão compartilhada com o paciente e familiares, valorizando as preferências do indivíduo e sua condição clínica, visando assim à maximização da qualidade de vida^9,17^.

Na literatura, estudos observacionais e randomizados realizados em diferentes regiões apontam que a HDD oferece sobrevida melhor ou similar à da HD realizada em clínicas^18,10,11,12,13,14,15,16,17,18,19,20,21,22,23,24^.Um estudo apontou sobrevida em um ano de 100%, em cinco anos de 83,5% e em 10 anos de 34,6% em pacientes em HDD com autopunção de fístula arteriovenosa, sendo a principal causa de morte a de origem cardiovascular^25^, semelhante ao observado em outros estudos^22,26,27,28,29,30,31,32,33,34,35,36,37,38,39,40,41^. Importante mencionar que nem todos os pacientes em diálise são candidatos a transplante renal. Além disso, um estudo prévio demonstrou sobrevida similar entre HDD e transplante renal com doador falecido de critério expandido^31^.

No Brasil, não há ainda estudos comparativos disponíveis entre a HDD e outras modalidades de Terapia Renal Substitutiva (TRS). No conjunto, os estudos que comparam desfechos envolvendo a HDD devem levar em consideração que pacientes elegíveis para a modalidade podem não ter o mesmo perfil clínico e socioeconômico daqueles tratados em centros de diálise, e esses dados devem ser examinados sob essa perspectiva.

A maioria dos estudos envolve a HDD de autocuidado, que é aquela realizada sem a presença de um profissional de saúde. No entanto, a HDD também pode ser feita com o acompanhamento desse profissional, embora os dados da literatura sejam escassos para essa modalidade.

Neste documento, a SBN desenvolve um posicionamento propondo requisitos mínimos para uma prática segura e efetiva de HDD no Brasil, tanto para o paciente quanto para o profissional de saúde, com o objetivo de colaborar para o alinhamento de políticas públicas, recursos e capacitação clínica dos centros de diálise.

## Métodos

### Etapas da Elaboração do Posicionamento da Sociedade Brasileira de Nefrologia Sobre Hemodiálise Domiciliar

#### Elaboração de Recomendações

As recomendações apresentadas neste documento foram elaboradas com base em evidências científicas, legislações brasileiras vigentes e opinião de especialistas. Participaram da elaboração do documento membros da Diretoria da SBN, do Departamento de Diálise da SBN, do Departamento de Defesa Profissional da SBN, além de nefrologista convidado com vasta experiência no gerenciamento de programas de HDD e de um representante do Comitê de Pacientes da SBN.

Situações que geraram possíveis divergências de opinião apresentaram dois perfis distintos: (i) de caráter técnico, nas quais foi possível embasar as recomendações em literatura especializada e normas vigentes; e (ii) de caráter gerencial, nas quais as sugestões foram elaboradas a partir da experiência dos autores e da realidade brasileira.

Após a redação inicial, o texto passou por um processo de revisão em duas etapas de consulta.

### Primeira Etapa da consulta

O documento preliminar foi submetido à apreciação da Diretoria da SBN, do Departamento de Diálise da SBN e do Departamento de Defesa Profissional da SBN. Simultaneamente, foi encaminhado às 19 Regionais ativas da instituição (Alagoas, Bahia, Ceará, Distrito Federal, Espírito Santo, Goiás, Maranhão, Minas Gerais, Mato Grosso, Pará, Paraíba, Paraná, Pernambuco, Piauí, Rio Grande do Norte, Rio Grande do Sul, Rio de Janeiro, Santa Catarina e São Paulo), com o objetivo de assegurar a representatividade das diversas realidades regionais do país.

#### Segunda Etapa da Consulta – Consulta Pública

As contribuições recebidas na primeira etapa foram analisadas e incorporadas quando pertinentes, mediante deliberação conjunta dos autores. Em seguida, a versão atualizada foi submetida à Consulta Pública, amplamente divulgada nas páginas eletrônicas e redes sociais da SBN. Paralelamente, foi encaminhada ao Conselho Federal de Medicina - CFM (SEI n^0^ 25.0.000006143-6) e à Associação Brasileira de Enfermagem em Nefrologia (SOBEN).

## Resultados

Na Consulta Pública, foram registradas 32 contribuições, das quais 14 (43,7%) manifestaram concordância integral, 14 (43,7%) concordância parcial e 4 (12,5%) discordância. Das 32 contribuições, foram sugeridos 42 itens de modificação do documento, dos quais 32 (76,1%) foram incorporados à versão final. As contribuições da Consulta Pública, com as respectivas considerações dos autores, estão disponíveis mediante solicitação à SBN e justificativa razoável. Os nomes dos autores das contribuições na Consulta Pública foram retirados, seguindo recomendação do Departamento Jurídico da SBN.

## Discussão

### Definição, Elegibilidades, Aspectos Técnicos do Tratamento e Treinamento da Hemodiálise Domiciliar

#### Definição e Tipos de Hemodiálise Domiciliar

A HDD é definida como a HD realizada no domicílio do paciente ou em instituições de longa permanência para idosos, onde o paciente reside.Os tipos incluem:

- **Autocuidado:** quando o paciente realiza o tratamento, com ou sem a ajuda de um familiar/cuidador, após treinamento intensivo e rigoroso (detalhado a seguir em “Treinamento intensivo do paciente para hemodiálise domiciliar de autocuidado”), e sem a presença de um profissional de saúde.
- **Assistida:** o paciente é submetido ao tratamento na presença de um profissional de saúde, que permanece no local durante todo o procedimento. O profissional requerido depende do perfil do paciente, conforme descrito na Figura 1.

**Figura 1:** Fluxograma do tipo de hemodiálise domiciliar conforme cenário clínico do paciente.

Abreviatura – HD: hemodiálise.

#### Critérios de Elegibilidade Para Hemodiálise Domiciliar

A admissão do paciente no programa de HDD deve respeitar os requisitos abaixo e basear-se na análise de elegibilidade realizada pela equipe multiprofissional (no mínimo pelo médico nefrologista, enfermeiro especialista em nefrologia, psicólogo e assistente social), com adequado registro em prontuário, e levando em consideração as crenças, os desejos e as expectativas do paciente e de seus familiares^38^.

#### Elegibilidade do Paciente Para Hemodiálise Domiciliar

- Paciente com DRC e indicação de TRS que opte pela modalidade de HDD, com concordância dos familiares e do médico nefrologista, com apoio da equipe multidisciplinar do centro de diálise (minimamente enfermeiro especialista em nefrologia, psicólogo e assistente social) e assinatura do Termo de Consentimento Livre e Esclarecido (sugestão no Anexo 1).
- Estar vinculado a um centro de diálise, que organizará consultas médicas no mínimo mensais (no centro ou no domicílio) para ajuste de prescrição médica.
- Condição clínica estável no centro de HD por no mínimo três meses, sem a ocorrência, durante as sessões, de complicações que constituam impedimentos à execução segura do procedimento.
- Acesso vascular para HD (admitindo-se apenas cateter tunelizado de longa permanência ou fístula arteriovenosa/prótese vascular) em boas condições, ou seja, sem evidência de infecção ou disfunção.
- Pacientes que já realizam autopunção (de fístula arteriovenosa ou prótese vascular) são potenciais bons candidatos ao método, desde que atendidos os critérios de elegibilidade e as demais avaliações necessárias.
- Pacientes com dificuldade de locomoção, temporária ou permanente, e/ou que residam em locais distantes do centro de diálise podem ser considerados para essa modalidade.

Caso o paciente deseje realizar HDD, mas não atenda aos critérios de elegibilidade (relacionados à condição clínica estável e à ausência de complicações durante a HD), sugerimos que seja claramente recomendada a HD em centro de diálise.

Na eventualidade de que qualquer um dos critérios acima se modifique ao longo do tempo, traduzindo risco excessivo, o paciente deve retornar ao tratamento no centro de diálise.

#### Elegibilidade Para Cada Tipo de Hemodiálise Domiciliar

Em conjunto com a equipe multiprofissional, o médico nefrologista deve definir, para cada paciente elegível para HDD, qual modalidade é recomendada: autocuidado ou assistida.

- **Para HDD de autocuidado:** a avaliação será realizada por meio de entrevista com o paciente, seus familiares e eventual cuidador, de forma a analisar as condições de aprendizado, a aceitabilidade e o interesse em relação a essa modalidade de HDD.
  - O paciente somente será avaliado quanto aos demais critérios de inclusão se toda a equipe multiprofissional concordar que ele seja, de fato, elegível, para então iniciar o treinamento.
  - O paciente deverá ter cognição preservada e boa aderência ao tratamento.
- **Para HDD assistida:** o paciente deverá apresentar condições clínicas, de entendimento e de motivação para essa modalidade, além de preencher os requisitos clínicos, sociais e domiciliares.
- Pacientes em situações especiais e temporárias podem ser candidatos à HDD assistida, conforme descrito no Quadro 1 e na Figura 1.

**Quadro 1:** Sugestões de tipo de hemodiálise domiciliar e profissional requerido, conforme cenário clínico do paciente

Modalidade de hemodiálise domiciliar	Perfil do paciente	Profissional de saúde presencial (mínimo)	Profissional à distância (mínimo)
Domiciliar de autocuidado	Paciente estável clinicamente, histórico de HD crônica em centro de diálise (satélite ou hospitalar) sem intercorrências, desejo de fazer HDD de autocuidado, elegível, avaliado e com treinamento intensivo e rigoroso para realizar o procedimento.	Nenhum	Médico nefrologista e equipe multidisciplinar
Domiciliar assistida	Paciente estável clinicamente, histórico de HD crônica em centro de diálise (satélite ou hospitalar) sem intercorrências graves, desejo de fazer HDD, avaliado e elegível. Sem desejo ou não apto para domiciliar de autocuidado.	Enfermeiro especialista em nefrologia	Médico nefrologista e equipe multidisciplinar
	Paciente temporariamente impossibilitado de ir até o centro de diálise (ex. fratura, imobilização), sem nenhuma outra mudança no quadro clínico.	Enfermeiro especialista em nefrologia	Médico nefrologista e equipe multidisciplinar
	Paciente em desospitalização, não elegível para retornar ao centro de diálise.	Enfermeiro especialista em nefrologia e médico nefrologista	Equipe multidisciplinar

Abreviaturas – HD, hemodiálise; HDD, hemodiálise domiciliar.

As indicações do perfil de paciente para cada tipo de terapia e o profissional requerido para assistência estão resumidas no Quadro 1 e na Figura 1.

De acordo com a legislação vigente, ressaltamos que a SBN não dispensa a presença do médico nefrologista na assistência presencial em centros de diálise, considerando ainda o perfil multicomórbido dos pacientes atendidos nesses ambientes. No que diz respeito à HDD, diante de qualquer mudança no cenário clínico, o paciente deverá ser reavaliado quanto à pertinência de mudança ou manutenção da modalidade, alterando-se, assim, a exigência do profissional presencial mínimo sugerido.

### Elegibilidade do Centro de Diálise de Referência

O centro de diálise que desenvolva programa de HDD deverá contar com estrutura física e dimensionamento de recursos humanos de acordo com a legislação vigente, e deverá também dispor de:

- Um espaço exclusivo para treinamento de HDD, separado dos demais pacientes em tratamento regular de HD, permitindo um treinamento mais efetivo.
- Prontuário individual do paciente, com registro da evolução do atendimento domiciliar pela equipe que o executou, preservando-se o acesso ao prontuário no centro de diálise.
- Manual com o detalhamento do programa de HDD (incluindo os Procedimentos Operacionais Padrão, os indicadores do programa, o modelo de treinamento do paciente e a sistemática de Educação Continuada dos profissionais), bem como a descrição da equipe assistencial e administrativa de suporte ao programa.

#### Elegibilidade do Domicílio

Antes do início da execução de HDD, deve ser realizada visita técnica domiciliar para garantir a viabilidade dessa atividade segundo as instalações físicas mínimas, conduzida ao menos pelo Responsável Técnico pela manutenção do tratamento de água do centro de diálise de referência, pelo enfermeiro especialista em nefrologista e pelo assistente social. A responsabilidade pela avaliação da elegibilidade do domicílio é do centro de diálise, que é habilitado e fiscalizado para tal pela Vigilância Sanitária (RESOLUÇÃO—RDC nº 917, DE 19 DE SETEMBRO DE 2024, que dispõe sobre o Funcionamento de Serviços que prestam Atenção Domiciliar).

- Deve-se avaliar os acessos domiciliares para recepção, fluxo e coleta de equipamentos e insumos.
- Podem ser necessárias a limpeza da caixa d’água e outras adequações para garantir a segurança do tratamento desse insumo. Deve-se avaliar seu suprimento, incluindo a verificação da temperatura da água da rede local e das condições do encanamento doméstico.
- Em alguns casos, são necessários ajustes na rede elétrica, que deve ser compatível com todos os equipamentos a serem utilizados e ser exclusiva do domicílio. Deve-se atentar ainda para a disponibilidade de meio de comunicação acessível para contato com a equipe do centro de diálise.
- A validação do domicílio pela equipe de enfermagem deve avaliar o espaço onde será executado o atendimento, com adequado planejamento da disposição dos equipamentos e insumos, do local onde permanecerão o paciente e os profissionais (no caso da HDD assistida), bem como da contingência de complicações intradialíticas que possam exigir medidas adicionais. O ambiente deve ser seguro, confortável do ponto de vista térmico, adequadamente iluminado e livre de ruídos excessivos, compatível com a limpeza regular das superfícies e com acesso facilitado a uma pia para higienização frequente das mãos. Em especial, animais domésticos não podem circular no ambiente de diálise, e deve-se limitar a circulação de crianças pequenas.
- Deve haver viabilidade de pronto atendimento por serviço de remoção de urgência e emergência durante o procedimento de HDD, garantindo o direcionamento do paciente para a rede hospitalar mais próxima, preferencialmente a de referência do centro de diálise.

#### Responsabilidades

- Os Responsáveis Técnicos (médico e enfermeiro especialista em nefrologia) do centro de diálise de referência manterão as mesmas responsabilidades, de acordo com as legislações vigentes, que possuem no centro de diálise, no que diz respeito à HDD.
- Os enfermeiros nefrologistas que realizam o treinamento devem ser profissionais dedicados e experientes (experiência mínima de seis meses em HD), competentes em todos os processos relevantes, no tratamento de água e na educação de adultos.
- O Responsável Técnico pelo tratamento de água do centro de diálise de referência tem como função tanto a verificação inicial da instalação elétrica e da rede de água potável quanto a verificação periódica dos equipamentos relacionados ao tratamento de água para HDD.
- O enfermeiro especialista em nefrologia e o médico nefrologista devem estabelecer, implantar e atualizar periodicamente Procedimentos Operacionais Padrão para cada fluxo de HDD: inclusão de paciente, checagem do domicílio, treinamento do paciente e/ou cuidador, entrega de material, controle de manutenção das máquinas, controle de resíduos, registros e guarda de prontuários e registros de atendimento.
- A prescrição da HDD é de responsabilidade do médico nefrologista, cabendo ao enfermeiro especialista em nefrologia a definição dos parâmetros específicos de sua atuação.
- O centro de diálise deve planejar e garantir a assistência ao paciente em caso de intercorrências relacionadas ao processo de HD, incluindo fluxos que assegurem a continuidade da atenção quando houver necessidade.
- O centro de diálise de referência é responsável pela logística da entrega de insumos e deslocamento de equipamentos, pela disponibilização de equipamentos de proteção individual (EPI) e pelo traslado de profissionais, respeitando-se, para isso, as respectivas legislações pertinentes. Há ainda a necessidade de elaboração de um Plano de Gerenciamento de Resíduos de Serviços de Saúde, com a descrição da sistemática de acondicionamento, segregação, armazenamento e destinação final (conforme a RDC 222, de 28/03/2018, e a RDC nº 917, de 19/09/2024), devendose, além disso, alinhar o plano de descarte de resíduos (perfurocortantes, biológicos e não-biológicos) com a Secretaria de Saúde local.
- O centro de diálise deve manter registros dos equipamentos e insumos utilizados no domicílio e ser responsável por apresentá-los, sempre que necessário, à vigilância sanitária, além de manter a rastreabilidade e o controle das manutenções preventivas.
- O serviço social e a psicologia devem avaliar a estrutura familiar, a autonomia do paciente e os recursos sociofamiliares disponíveis em caso de necessidade^39^, assim como o impacto da implantação da HD no domicílio e na vida familiar (tanto no que diz respeito à compreensão do que costuma ocorrer quanto aos eventuais impactos financeiros, como o maior consumo de água e luz)^23^.

#### Como Realizar a Hemodiálise Domiciliar

- Qualquer máquina de HD ou hemodiafiltração (HDF) devidamente registrada na ANVISA e com controle de ultrafiltração poderá ser usada; aguardam-se ainda, no Brasil, equipamentos e insumos de menor porte para uso domiciliar.
- O tratamento de HDD permite maior flexibilidade da prescrição, com potencial emprego de maior frequência e/ou maior duração da sessão. A escolha da frequência e da duração das sessões de hemodiálise deve ser baseada em avaliação clínica criteriosa e na prescrição médica, considerando parâmetros de adequação dialítica, condições individuais do paciente e suas preferências, dentro de um processo de decisão compartilhada.
- A prescrição médica de HDD (especialmente na escolha dos fluxos de sangue e de dialisato, na frequência das sessões e na duração do procedimento) deve definir os padrões a serem executados^42,43,44^. Ressalta-se que: (1) o aumento do número semanal de horas de HD está associado à melhora da sobrevida; (2) HD mais frequente e mais longa permite menores taxas de ultrafiltração, com menor risco de instabilidade hemodinâmica, isquemia tecidual, arritmias e morte súbita; e (3) evitar um intervalo prolongado sem HD está associado à melhora da sobrevida^6,45,46,47^.
- A periodicidade da coleta e os tipos de exames laboratoriais devem seguir, como requisito mínimo, a periodicidade estabelecida pela normativa aplicável. Deve ser dada atenção à hipofosfatemia, que é comumente verificada em HDD frequente e de longa duração^48^.
- O sistema extracorpóreo de HD (filtros e linhas) não pode ser reprocessado, devendo ter destino específico após o uso (conforme a RDC 222, de 28/03/2018).
- Para HDD assistida, cabe ao enfermeiro especialista em nefrologia avaliar sistematicamente o estado geral, as queixas, a pressão arterial, a frequência cardíaca e a temperatura do paciente; na presença de alterações, o mesmo deve comunicar o médico antes de iniciar a sessão (incluindo: pressão arterial sistólica – PAS < 90 mmHg; PAS > 180 mmHg; taquicardia/bradicardia; hipo/hipertermia; alteração do estado geral; ou queixas clínicas).
- Aferição da pressão arterial: para HD de até quatro horas e fora do horário de sono do paciente, recomenda-se medir a pressão arterial a cada 30 a 60 minutos durante a sessão. Para pacientes em HDD de autocuidado realizada durante a noite, não há exigência de medida de pressão arterial a cada 30 minutos, sugerindose, no mínimo, a aferição no início e ao final da sessão.
- O paciente (no caso de HDD de autocuidado) ou o profissional de saúde deverão manter, em todas as sessões de diálise, o registro de: pressão arterial, ultrafiltração, peso pré e pós-HD, hora de início e término da terapia, intercorrências ocorridas e respectivas condutas. As medidas obtidas de pressão arterial e frequência cardíaca devem ser armazenadas no prontuário do paciente.
- Recomenda-se que o telemonitoramento à distância (realizado pela equipe do centro de diálise de referência do paciente) ocorra regularmente e sempre que necessário ou desejável. No caso de HDD de autocuidado, sugere-se que seja realizado pelo menos nas primeiras duas semanas de tratamento. Após esse período, o paciente deve manter acompanhamento com equipe multidisciplinar e ser avaliado conforme demanda individualizada, além do atendimento já programado.
- Atendimentos remotos do paciente pelo médico nefrologista, em casos de urgência/emergência e com impedimento demonstrável de assistência presencial, são válidos de acordo com o artigo 37 do capítulo V do Código de Ética Médica de 2019. Além disso, a regulamentação da teleconsulta no Brasil está estabelecida pela Lei nº 14.510/22 e pela Resolução CFM nº 2.314/2022.
- É necessária a definição prévia de retaguarda em serviço de urgência/emergência, não sendo recomendada a realização de HDD em locais onde esse suporte não esteja disponível. O atendimento e o transporte/transferência em emergências devem ser realizados pelo Serviço de Atendimento Móvel de Urgência (SAMU) ou por serviço de ambulância contratado, preferencialmente com tempo médio de resposta de 30 minutos.
- Consultas ambulatoriais devem ser programadas, no mínimo, mensalmente e são ocasiões em que o paciente terá acesso ao médico nefrologista, ao enfermeiro especialista em nefrologia, ao psicólogo, ao assistente social e ao nutricionista.
- A qualquer momento em que o paciente desejar, ou quando for julgado necessário pela equipe assistencial, será possível o retorno ao centro de diálise para reavaliação e, se indicado, a realização de novo treinamento. Caso haja necessidade clínica ou desistência da modalidade domiciliar, o centro de diálise deverá prever mecanismos que viabilizem o retorno do paciente à HD no centro, dentro da disponibilidade de vagas e com prioridade compatível com a situação clínica.
- Em caso de dúvidas, o paciente deve manter contato, por telefone, com o enfermeiro especialista em nefrologia ou com o médico nefrologista no centro de diálise, no horário habitual de funcionamento deste. O treinamento do paciente deverá incluir a entrega de um cartão com os telefones do centro de diálise.
- Em caso de problemas com a máquina de diálise que não possam ser solucionados a tempo da próxima sessão, o paciente poderá realizar o tratamento de HD no centro, em horário a ser combinado com o responsável pelo setor de agendamento.
- Visitas regulares à casa do paciente devem ser programadas mensalmente, ou mais amiúde, de acordo com a necessidade, pelos técnicos responsáveis pelo tratamento da água e pela manutenção da máquina de HD.
- Devem estar definidas as medidas destinadas a garantir a segurança do paciente na modalidade de HDD^8^, incluindo a incorporação e manutenção da Cultura da Segurança^49^, o estabelecimento de barreiras para impedir eventos^50^ e a investigação e o tratamento de incidentes pelo Núcleo de Segurança do Paciente do centro de diálise responsável pelo tratamento^51^.
- As recomendações pertinentes às imunizações devem estar em conformidade com a Política Nacional de Imunizações^52^ do Ministério da Saúde.
- O atendimento de pacientes com hepatite B segue a mesma rotina prevista na legislação vigente, recomendando-se que seja realizado com máquina dedicada exclusivamente ao paciente e por trabalhadores da saúde com níveis de anticorpos protetores (anti-HBs).
- Sugere-se o gerenciamento sustentável^53,54^ da água de rejeito (compatível com uso doméstico para irrigação de jardins, limpeza geral, lavagem de roupas e descarga em vasos sanitários) e uma cuidadosa destinação do efluente dialítico, em conformidade com a legislação.
- O descarte do material de HDD deve respeitar as mesmas normas aplicáveis aos centros de diálise satélites, com descarte de linhas e filtros como material biológico. Perfurocortantes devem ter local e destinação de descarte apropriados. Recomenda-se, ainda, que no treinamento da modalidade de HDD o paciente seja esclarecido sobre onde o descarte ocorre e como o lixo é transportado. Em nenhuma hipótese o lixo deve ser descartado como lixo comum de domicílio.

#### Treinamento Intensivo do Paciente Para Hemodiálise Domiciliar de Autocuidado

O espaço físico necessário para iniciar o treinamento do paciente poderá ser o mesmo ambiente em que ele realiza sua HD de rotina, mas favorecendo a maior privacidade.

Será explicado ao paciente como ocorrerá o processo de tratamento de HD em sua residência e que, diante da ocorrência de qualquer dificuldade (seja com insumos ou com a empresa envolvida), ele terá direito de retornar ao seu tratamento de HD no centro de referência.

O paciente será treinado para procedimentos referentes à própria HD: preparar, ligar e desligar a máquina, entender e resolver os diversos alarmes do equipamento, aplicar procedimentos de segurança e realizar a punção da fístula arteriovenosa ou a conexão do cateter.

O paciente será acompanhado e treinado, desde o início, pelo mesmo enfermeiro, no horário determinado, conforme sua rotina de terapia. O treinamento compreende um período mínimo de dois meses ou 24 sessões, podendo ser estendido conforme necessário. Uma pessoa de contato domiciliar será capacitada para reconhecer alarmes e eventos adversos^50^ e executar os procedimentos pertinentes; esse cuidador poderá, ainda, receber treinamento integral juntamente com o paciente.

O treinamento do paciente (e do cuidador) para HDD será composto de estágios:

1. O componente inicial consiste em uma introdução e orientação ao paciente, abordando independência e responsabilidade com o tratamento, além de cuidados com o acesso vascular e da importância de seguir estritamente a rotina de segurança em casa.
2. As técnicas de ensino utilizadas durante o treinamento são de responsabilidade do centro de diálise e podem incluir exemplos práticos, demonstrados repetidamente. Quando usado durante todo o período de treinamento, esse processo desenvolve a confiança na utilização da máquina de HD ou HDF, na solução de problemas indicados pelos alarmes e na montagem e desmontagem do material necessário para a diálise, permitindo que o paciente aprenda a autopunção do acesso ou a conexão do cateter venoso central com segurança, capacitando-o ainda a descontinuar o tratamento, usar a tecnologia de desinfecção da máquina e realizar o tratamento da água.
3. O treinamento inclui a resolução de problemas que possam ocorrer à noite e os protocolos de emergência, incluindo o chamado de emergência para o 192 (ou ambulância privada, no caso de pacientes atendidos por operadoras da Saúde Suplementar, não-SUS) ou a ida ao prontosocorro mais próximo de sua residência.
4. Um manual contendo todo o conteúdo explicado durante o treinamento deverá ser entregue ao paciente, no qual se revisam os cuidados para iniciar, manter e terminar o tratamento, além da resolução de alarmes e de possíveis procedimentos de emergência.
5. Durante o período de treinamento, devem ser aplicados três testes escritos para assegurar que o paciente e/ou cuidador estejam aptos a realizar os procedimentos com segurança. Esses testes escritos são recursos para formalizar a efetividade do treinamento, de forma a caracterizar que o paciente e/ou cuidador saibam como devem proceder diante das diversas situações, sejam elas de risco ou não, que possam ocorrer em casa. Sugere-se uma nota mínima de 70 a 90% de acertos nesses testes (à critério do centro de diálise). O treinamento deve abranger tópicos definidos, habilidades de resolução de problemas e avaliações baseadas em competências.
6. Ao final do período de treinamento, espera-se que o paciente e/ou cuidador possam realizar todo o processo de HD sem nenhuma ajuda da enfermagem (que permanece somente observando, na sala de diálise onde ocorre o treinamento). Essa etapa de segurança garante que toda a equipe possa observar o paciente e/ou cuidador como se estivessem em uma sessão de HDD. A duração desse período poderá variar de paciente para paciente.
7. Após o período de treinamento, pretende-se que o paciente e/ou cuidador possam realizar na prática todos os procedimentos, sendo então liberados pelo médico nefrologista e pelo enfermeiro especialista em nefrologia para iniciar o tratamento em casa. Na eventualidade de alguma insegurança na execução do procedimento, seja do ponto de vista do enfermeiro, do médico ou do próprio paciente e/ou cuidador, o paciente deverá ter seu tratamento dialítico ajustado para outra modalidade de diálise mais apropriada a cada contexto.
8. O paciente deverá ser orientado a trazer, nas consultas minimamente mensais, uma planilha de registros (fornecida pela equipe) contendo as informações da HDD, como peso, pressão arterial e programação da taxa de ultrafiltração realizada por ele. É de responsabilidade do paciente apresentar esse documento de registro nas consultas mensais, para posterior arquivamento no prontuário médico.

## Aspectos Técnicos da Qualidade da Água, Análise Sobre Custo e Aspectos Legais da Hemodiálise Domiciliar

### O Tratamento da Água Para Hemodiálise Domiciliar

O tratamento da água deve obedecer aos parâmetros estabelecidos na legislação vigente. O centro de diálise de referência para o programa de HDD deve manter os laudos das análises de água disponíveis para apresentação à Vigilância Sanitária.

- Em conformidade com a RDC ANVISA nº 11, de 13/03/2014, a água de abastecimento do domicílio a ser tratada com o equipamento de osmose reversa portátil (ORP) deve apresentar padrão de potabilidade de acordo com a legislação vigente (Art. 45). Suas características físicas e organolépticas (cor aparente, turvação, sabor, odor, cloro residual livre e pH) devem ser aferidas antes de cada sessão de HDD (conforme Quadro I do anexo da referida RDC), com registro em prontuário.
- A água produzida pela ORP deve atender aos mesmos padrões previstos para a qualidade da água (conforme Quadro II do anexo da RDC ANVISA nº 11, de 13/03/2014, que estabelece os componentes de monitorização mensal e semestral), devendo as análises ser realizadas por laboratório analítico licenciado pelo órgão sanitário competente.

Em detalhes, recomenda-se:

- Limpeza da caixa d’água no momento inicial e a cada seis meses;
- Osmose: sanitização a cada 30 dias em caso de HD e a cada 15 dias em caso de HDF;
- Teste diário de cloro da água antes do início da sessão de HD;
- Análise mensal da água para exame microbiológico;
- Análise semestral da água para exame físico-químico;
- Análise anual do dialisato;
- Análise anual da água potável;
- Manutenção dos equipamentos para possíveis trocas de válvula, bomba, engates e mangueiras, se necessário, e sempre que houver falha no equipamento.

No caso de edificações de uso multifamiliar, como apartamentos e condomínios, a contratação e supervisão da limpeza dos reservatórios (caixa d´água) por empresa especializada é atribuição do síndico. Além da rotina inicial e semestral, limpezas adicionais são mandatórias em situações de potencial contaminação (rompimento de adutoras, ingresso de detritos, entre outras). Sugere-se reunião prévia de alinhamento com o síndico e, se necessário, com a presença do médico nefrologista, do enfermeiro especialista em nefrologia e/ou do responsável técnico pelo tratamento de água do serviço de diálise.

### Custo e Financiamento da Hemodiálise Domiciliar

O custo deverá incluir o uso de materiais essenciais e máquinas de diálise e de osmose. Os materiais são os mesmos utilizados em centros de diálise e incluem filtros e conjunto de linhas de sangue arterial e venosa de uso único, soluções de diálise ácida e básica, isoladores de pressão, agulhas de fístula, sanitizante para desinfecção da máquina, filtro usado no processo de desinfecção, soro fisiológico, gazes, heparina, luvas e seringas.

O ressarcimento pelas fontes pagadoras—tanto no sistema público quanto na saúde suplementar—deve primar pelo equilíbrio entre custo e benefício para os provedores e os centros de diálise, respeitando as condições e necessidades locais. A discussão de modelos de financiamento é fundamental para viabilizar a HDD no Brasil. Deve haver definição clara da elegibilidade e dos requisitos a serem atendidos para preservar qualidade e segurança, com o devido dimensionamento dos custos e a definição adequada dos valores a serem reembolsados.

### Aspectos Legais da Hemodiálise Domiciliar

Atualmente, o Brasil não dispõe de regulamentação específica para HDD, como uma RDC ou Portaria do Ministério da Saúde, que defina padrões específicos de elegibilidade, execução, avaliação e monitoramento dessa modalidade terapêutica.

Na Resolução que dispõe sobre o Funcionamento de Serviços que prestam Atenção Domiciliar (Resolução– RDC nº 917, DE 19 DE SETEMBRO DE 2024), lê-se no Art. 25 que o Serviço de Atenção Domiciliar (SAD), de acordo com o Plano de Atenção Domiciliar (PAD), deve assegurar a terapia de diálise, e que, na realização de hemodiálise, o dialisador deve ser de uso único.

Portanto, este posicionamento da SBN tem por propósito estabelecer parâmetros preliminares para a modalidade de HDD em nosso país, preservando a qualidade e a segurança do procedimento, assim como a atuação dos profissionais envolvidos, além de alinhar a HDD com a legislação pertinente à especialidade. A legislação vigente, no âmbito da ANVISA e do Ministério da Saúde, deverá sofrer ajustes no sentido de prever a realização domiciliar de hemodiálise e estipular boas práticas para sua execução.

## Conclusão

A adesão à modalidade de HDD deve ser, portanto, objeto de reflexão conjunta entre equipe, paciente e familiares, resultando em plano terapêutico formal e na assinatura de um termo de consentimento livre e esclarecido, no qual se declare a escolha autônoma pela modalidade e se delimitem as expectativas e responsabilidades de cada envolvido.

O presente documento traz recomendações técnicas e operacionais que visam estabelecer bases para a prática segura, eficaz e factível da modalidade no Brasil. Ao reunir aspectos clínicos, logísticos, legais e de sustentabilidade, esta posição da Sociedade Brasileira de Nefrologia pretende subsidiar pactuações entre gestores e executores, tanto no SUS quanto na saúde suplementar, contribuindo para a ampliação do acesso e para a consolidação de políticas alinhadas com padrões internacionais de qualidade em diálise.

## Data Availability

As contribuições da Consulta Pública e as respectivas considerações dos autores podem ser obtidas mediante solicitação formal à Sociedade Brasileira de Nefrologia (SBN), acompanhada de justificativa razoável. Por orientação do Departamento Jurídico da SBN, os nomes dos autores das contribuições foram suprimidos.

## Material Suplementar

O seguinte material online está disponível para o presente artigo:

Anexo 1 Modelo de termo de consentimento livre e esclarecido para hemodiálise ou hemodiafiltração domiciliar.
